# Diffusion Tensor Imaging Features of the Auditory Pathways in Patients With Vestibular Schwannoma After Gamma Knife Radiosurgery

**DOI:** 10.7759/cureus.14143

**Published:** 2021-03-27

**Authors:** Dilek H Cesme, Alpay Alkan, Mehmet Ali Gultekin, Lutfullah Sari, Gokberk Alkan, Ahmet Kaya, Alpaslan Mayadagli, Kerime Akdur, Omer Uysal, Mustafa A Hatiboglu

**Affiliations:** 1 Department of Radiology, Faculty of Medicine, Bezmialem Vakif University, Istanbul, TUR; 2 Otorhinolaryngology, Abdurrahman Yurtaslan Oncology Training and Research Hospital, Ankara, TUR; 3 Radiation Oncology, Faculty of Medicine, Bezmialem Vakif University, Istanbul, TUR; 4 Biostatistics, Faculty of Medicine, Bezmialem Vakif University, Istanbul, TUR; 5 Neurosurgery, Faculty of Medicine, Bezmialem Vakif University, Istanbul, TUR

**Keywords:** vestibular schwannoma, auditory pathways, gamma knife radiosurgery, radiosurgery, diffusion tensor imaging

## Abstract

Objective

In this study, we aimed to investigate whether there is any change in diffusion tensor imaging (DTI) parameters in ipsilateral and contralateral auditory pathways after Gamma Knife radiosurgery (GKR) in patients with vestibular schwannoma (VS) and the relationship between radiosurgery variables.

Methods

Sixty-six patients were evaluated with MRI and DTI before and after GKR. The apparent diffusion coefficient (ADC) and fractional anisotropy (FA) were measured from the bilateral lateral lemniscus (LL), inferior colliculus (IC), medial geniculate body (MGB), and Heschl's gyrus (HG).

Results

There was no significant difference in ADC and FA values obtained from bilateral LL, IC, and MGB before and after radiosurgery. However, there was a significant difference between pretreatment and post-radiosurgery contralateral HG ADC values. The ADC values obtained from the contralateral HG and IC positively correlated with the duration after radiosurgery. As the duration after radiosurgery increases, the difference between the ADC values obtained from ipsilateral and contralateral HG also increases.

Conclusion

The high ADC values in the contralateral HG after radiosurgery may indicate microstructural alterations such as demyelination and axonal loss. Radiation exposure doses to the brainstem and cochlea are the most important factors that can cause microstructural damage to the auditory pathways. When planning radiosurgery, extreme care should be taken to prevent the harmful effects of radiation on the auditory pathways.

## Introduction

Vestibular schwannomas (VS) are benign neoplasms of Schwann cell origin. The most common symptom of the condition is unilateral neurosensory hearing loss. Additionally, dizziness, tinnitus, imbalance, headache, paresthesia, and facial paralysis complaints are also seen [1,2].

Treatment options for VS include observation, microsurgery, or radiosurgery [2-4]. Currently, Gamma Knife radiosurgery (GKR) is the preferred treatment method for small and medium-sized tumors. The aim of radiosurgery treatment is to permanently restrain the growth of VS [5]. Brainstem and cochlear radiation dose parameters have critical importance in planning radiosurgery treatment. The higher the radiation dose to which the brainstem is exposed, the greater the risk of hearing loss [6-11]. Hearing loss develops due to the toxic effect of radiation in the brainstem nuclei and cochlea [6,7,11]. When planning stereotactic radiosurgery for VS, especially for large lesions, the cochlear nerve will always be involved as it cannot be separated from the superior and inferior vestibular.

The auditory pathways consist of the lateral lemniscus (LL), inferior colliculus (IC), medial geniculate body (MGB), and Heschl's gyrus (HG). Fibers from the brainstem cochlear nuclei extend into the ipsilateral or contralateral superior olivary nucleus and reach the IC via the LL. Efferent fibers extend from the IC through the MGB in the thalamus to the auditory cortex in the HG [12,13].

Diffusion tensor imaging (DTI) is an advanced neuroimaging method used to investigate white matter microstructure integrity and connectivity [6]. Fractional anisotropy (FA) and apparent diffusion coefficient (ADC) and are the most frequently used parameters in DTI. FA values provide important data about myelination, fiber density, and axon diameter in white matter. The decrease in FA may occur due to the loss of regular anisotropic structures, possibly due to a disruption of the cytoarchitecture. While ADC independently measures the direction of total water diffusion in tissues, it also provides information about tissue cellularity and nucleus-cytoplasm ratio. Increased ADC values are thought to be associated with increased extracellular fluid production and myelin disorders due to a decrease in the number of cells or axons [6,14-16].

DTI studies that evaluate auditory pathways such as LL, IC, MGB, and HG in patients with VS treated with radiosurgery are scarce [6]. In this study, we aimed to evaluate whether there is any change in DTI parameters in auditory pathways after radiosurgery in patients with VS and to investigate the correlation between radiosurgery variables and DTI parameters.

## Materials and methods

Our study was approved by the Bezmialem Vakıf University Clinical Research Ethics Committee (54022451-050.05.04/2020). We retrospectively collected the data of the subjects who underwent GKR treatment for VS between 2013 and 2020 at our University Hospital. Sixty-six patients (40 males, 26 females; mean age: 54.65 ±13 years; age range: 30-77 years) with VS who underwent MRI and DTI imaging before and after treatment were included in the study. Patients with a history of microsurgical operation in the cerebellopontine angle and those without a follow-up MRI after radiosurgery were excluded.

Magnetic resonance imaging

The subjects with unilateral VS who were considered for GKR treatment were evaluated with a 1.5T MRI system (Avanto, Siemens Healthineers, Erlangen, Germany). The applied MRI sequences included axial and coronal fluid-attenuated inversion recovery (FLAIR) [repetition time (TR): 8000 ms, echo time (TE): 90 ms, inversion time (TI): 2500 ms], sagittal, axial T2 turbo spin-echo (TSE) (TR: 4500 ms, TE: 90 ms), and axial T1 spin-echo (SE) (TR: 550 ms, TE: 14 ms) weighted images. T1 images with contrast in the axial, sagittal, and coronal planes were obtained [IV gadolinium-diethylenetriaminepentaacetic acid (Gd-DTPA)]. 3D T1 magnetization-prepared rapid acquisition with gradient echo (MPRAGE) images with and without contrast were added to our study.

All subjects were evaluated with DTI parameters as standard protocol [single-shot SE echo-planar, TR/TE: 6000/89 ms, field of view (FOV): 230 x 230 mm, matrix: 128 x 256, and thickness: 5 mm]. Thirty different diffusion gradient directions were used (b=0 s/mm^2^ and b=1000 s/mm^2^). ADC and FA maps were created by processing DTI data on a workstation (Leonardo console, software version 2.0; Siemens). Axial color-encoded FA map images were taken as a reference while placing the regions of interest (ROI) in the auditory pathways. Four pixel ROI sizes were placed manually. Placement of all ROIs was performed simultaneously by an experienced radiologist. Follow-up images were obtained 3-60 (19.8 ±15.5) months after the baseline images taken before radiosurgery.

Gamma Knife protocol

**Figure 1:**
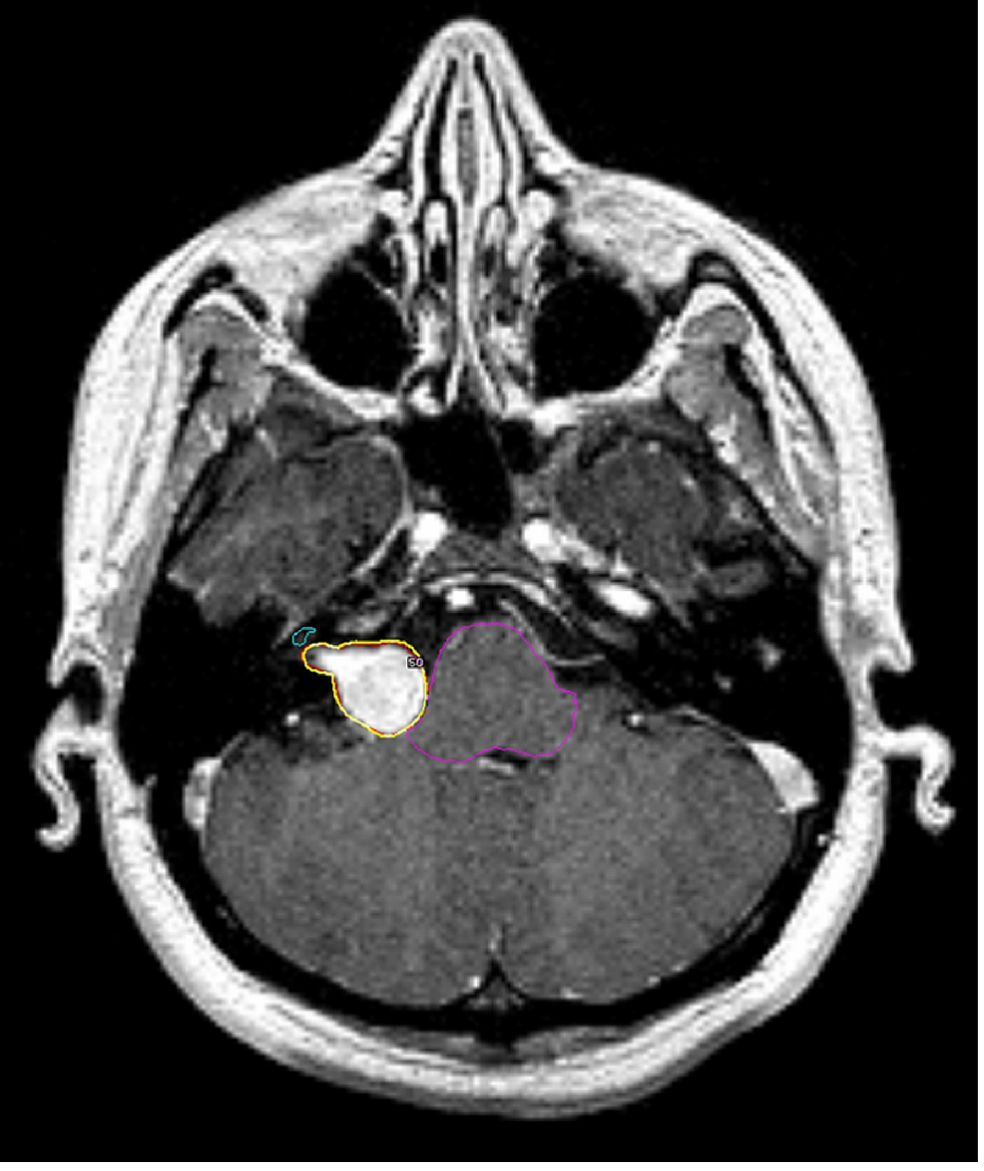
Radiosurgery planning in a case of vestibular schwannoma The boundaries of the brainstem and cochlea (colored outlines) were defined to calculate the exposure dose

**Figure 2:**
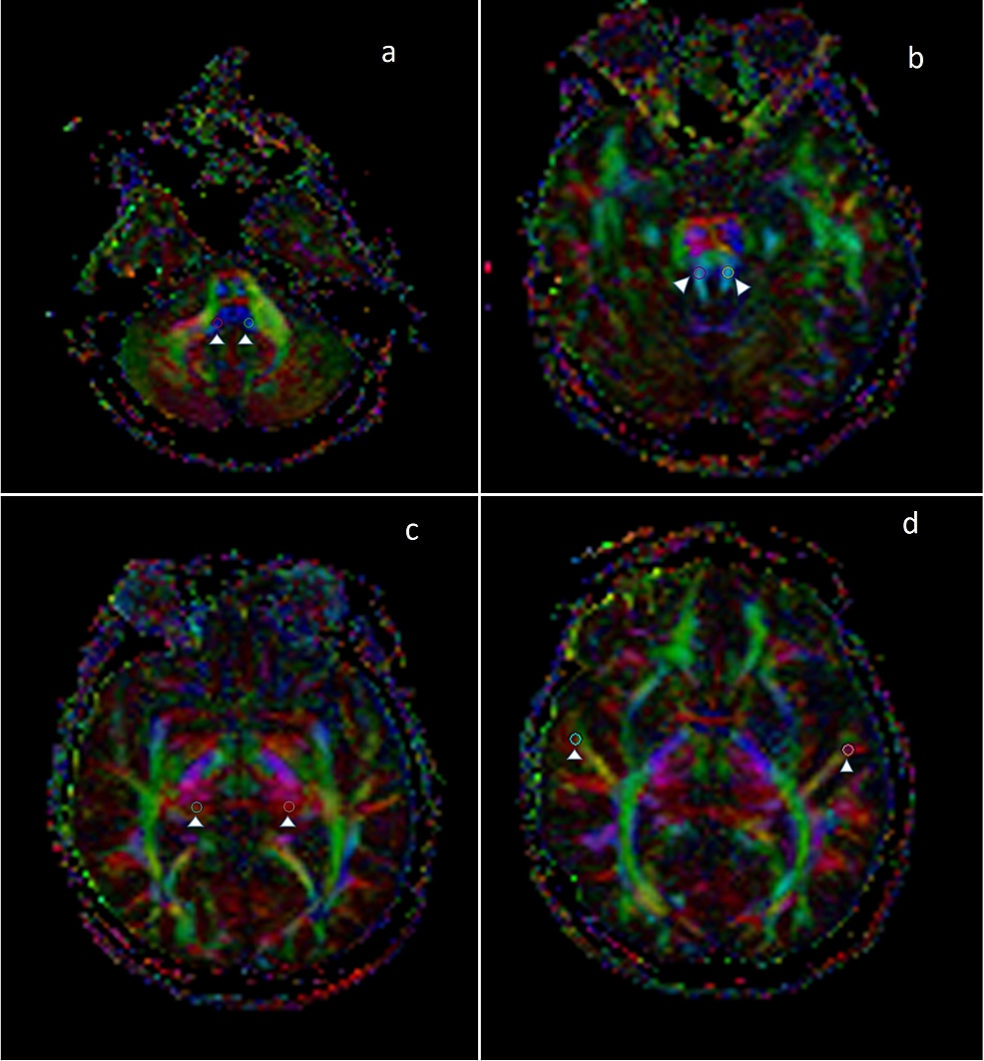
Right-sided vestibular schwannoma The color FA maps show ROI placement at lateral lemniscus (a) (arrowheads), inferior colliculus (b) (arrowheads), medial geniculate body (c) (arrowheads), and Heschl's gyrus (d) (arrowheads) FA: fractional anisotropy; ROI: region of interest

Treatment planning was carried out using the Gamma Knife 4C model and its software version 10.1.1 (Elekta, Stockholm, Sweden). A Leksell stereotactic head frame was placed, and images of volumetric MRI sequences 3D T2 and 3D T1 MPRAGE were taken. All patients were treated with an isodose of 50%: the median treatment dose was 12.5 Gy (range: 11.5-13 Gy), the median maximum brainstem dose was 10.4 Gy (range: 0.7-15.1 Gy), the median brainstem volume receiving a radiation dose of 10 Gy (BS V10) was 0.1 cc (range: 0-0.55 cc), the median maximum cochlear dose was 5.8 Gy (range: 1.4-12.1 Gy), and the mean cochlear dose was 3.1 Gy (range: 0.9-8.3 Gy) (Figure 1). No additional neurological deficits were detected in the follow-up MRIs after radiosurgery. The ADC and FA values of LL, IC, MGB, and HG were measured before and after radiosurgery (Figures 2a-2d). Moreover, the relationship between radiosurgery treatment variables such as the brainstem and cochlear dose and ADC and FA values ​​was investigated.

Statistical analysis 

Statistical data were evaluated using SPSS Statistics v19.0 (IBM, Armonk, NY). The Kolmogorov-Smirnov test was used to determine whether the data were suitable for normal distribution. Parametric tests were applied since they show normal distribution. A paired t-test was used to compare the FA and ADC values ​​obtained from the auditory pathways before and after radiosurgery. Pearson's correlation test was used to investigate the relationship between DTI parameters obtained from auditory pathways and GKR treatment variables. A p-value of <0.05 was considered statistically significant.

## Results

There was a significant difference in LL FA values between the ipsilateral and contralateral sides in the pretreatment period (p=0.001). Contralateral LL FA values were lower than ipsilateral ones in the pretreatment period. However, there was no significant difference in ADC and FA values obtained from bilateral LL, IC, and MGB before and after radiosurgery.

There was a significant difference between pretreatment and post-radiosurgery contralateral HG ADC values (p=0.002). The ADC values obtained from the contralateral HG and IC positively correlated with the duration after radiosurgery (r=0.265, p=0.03 and r=0.241, p=0.05, respectively). As the duration after radiosurgery increased, the difference between the ADC values obtained from ipsilateral and contralateral HG also increased (r=0.282, p=0.02).

As the maximum brainstem radiation dose increased, the difference between the contralateral HG FA values before and after radiosurgery also increased (r=0.247, p=0.04). Ipsilateral MGB ADC values ​​were positively correlated with the maximum cochlear dose (r=0.300, p=0.01) and the mean cochlear dose (r=0.269, p=0.02).

DTI parameters obtained from the auditory pathways before and after radiosurgery are presented in Table 1.

**Table 1:** The ADC and FA values measured before and after radiosurgery from ipsilateral and contralateral sides in patients with VS LL: lateral lemniscus; IC: inferior colliculus; MGB: medial geniculate body; HG: Heschl’s gyrus; GKR: Gamma Knife radiosurgery; ADC: apparent diffusion coefficient (10^-3^ mm^2^/sec); FA: fractional anisotropy; VS: vestibular schwannoma

Locations		ADC			FA		
		Pre-GKR	Post-GKR	P-value	Pre-GKR	Post-GKR	P-value
LL	Ipsilateral	0.778 ±0.75	0.777 ±0.61	0.98	0.620 ±0.10	0.616 ±0.10	0.79
	Contralateral	0.771 ±0.63	0.789 ±0.75	0.10	0.581 ±0.11	0.598 ±0.13	0.21
IC	Ipsilateral	0.783 ±0.58	0.777 ±0.63	0.75	0.680 ±0.10	0.708 ±0.12	0.06
	Contralateral	0.780 ±0.60	0.777 ±0.74	0.72	0.674 ±0.11	0.707 ±0.13	0.06
MGB	Ipsilateral	0.783 ±0.59	0.797 ±0.85	0.35	0.448 ±0.85	0.436 ±0.85	0.39
	Contralateral	0.785 ±0.62	0.795 ±0.75	0.38	0.441 ±0.88	0.442 ±0.95	0.93
HG	Ipsilateral	0.795 ±0.92	0.809 ±0.68	0.36	0.299 ±0.75	0.282 ±0.96	0.27
	Contralateral	0.772 ±0.69	0.806 ±0.70	0.002	0.316 ±0.90	0.307 ±0.83	0.58

## Discussion

VS accounts for approximately 6-8% of all brain tumors. The main goal of the treatment of VS should be functional preservation of the facial, trigeminal, and cochlear nerves rather than complete removal of the tumor. Radiosurgery is the primarily preferred treatment method as it is associated with over 95% remission, low complication rate, preservation of neurological functions, and good quality of life in patients [5,17-20].

Hearing stimuli originate from the cochlear branch of the eighth cranial nerve. The ventral and dorsal cochlear nuclei located in the brainstem are very important anatomical functional structures for auditory pathways. The postsynaptic fibers are called LL. All of the fibers initiating from the cochlear nuclei are not crossed but a few of them ascend as ipsilateral lemniscus lateralis. These fibers then intermingle into the IC, the center of hearing reflexes in the mesencephalon. The fibers starting from the IC then reach the auditory cortex (HG) after synapse at MGB. The contralateral pathways have a much more important function in the processing of auditory stimuli. Also, 20-30% of the fibers that reach the auditory cortex come from the ipsilateral side and 70-80% come from the contralateral side [12,13].

DTI is a promising imaging technique used to characterize microstructural changes or differences in neuropathological events [6,12,14-16]. It can enable the identification of auditory neural tracts. Decreased FA values may suggest white matter damage and axonal degeneration. It is considered that increased ADC values ​​may be associated with increased extracellular fluid production and myelin disorders due to a decrease in the number of cells or axons [21]. ADC values may be affected in tissue damage such as edema, degeneration, and necrosis [21]. Radiation-induced changes in the auditory pathways can be detected in patients with VS treated with radiosurgery [6,8,9,22]. In our study, contralateral LL FA values were lower than ipsilateral in the pretreatment period. The low FA values in the contralateral LL can be attributed to the microstructural alterations in the white matter tract depending on the anatomical course of the auditory pathway in the pre-radiosurgery period.

DTI studies showing diffusion changes in auditory pathways in various diseases accompanied by neurosensory hearing loss are available in the literature [6,12,14-16]. In a DTI study performed in patients with unilateral VS, ADC values increased ​​in the contralateral LL, IC, and MGB compared to healthy controls [12]. The authors suggested that high ADC values on the contralateral side may be associated with microstructural changes such as demyelination. They explained that the increase in ADC values on the contralateral side may be due to the natural anatomical course of the auditory pathways [12]. They also found low FA values on both sides in IC. They reported that decreased FA values may be associated with axonal loss, decreased fiber density, and/or demyelination [12]. The changes in ADC and FA values may be attributed to axonal loss and/or demyelination as a result of prolonged injury to the auditory pathways. In another study, the authors reported that ADC and FA values ​​in auditory pathways did not change after radiosurgery in patients with VS [6]. They associated it with the presence of the tumor affecting the auditory pathways before radiosurgery. In the current study, we did not detect any changes in ADC and FA values ​​after radiosurgery at LL, IC, and MGB. Our results indicate that auditory pathways may have been influenced by the presence of VS prior to the treatment.

ADC values in contralateral HG increased significantly after radiosurgery. ADC values that increase depending on the delay after radiosurgery may be explained by microstructural alterations such as demyelination. We speculate that the presence of increased ADC values in the contralateral HG may be correlated to the anatomical course of the auditory pathway fibers. Our results support the hypothesis that stimuli from the contralateral side contribute to the processing of auditory stimuli in HG to a greater extent [6,12]. In addition, contralateral IC ADC values increase depending on the time after radiosurgery. The presence of increased ADC values at the IC level can be ascribed to the reception of more than one stimulus from the lower auditory pathways on both sides of the brain [5].

The most important parameters in estimating radiation toxicity to the brainstem in radiosurgery treatment are the maximum brainstem dose and BS V10 [14]. In our study, as the radiation dose exposed to the brainstem increased, the contralateral HG FA values decreased slightly. There was no strong correlation between them. A decrease in FA values may be associated with alterations in cytoarchitecture [22]. The weak relationship between the brainstem radiation dose and the slight decrease in contralateral HG FA values may indicate that no significant permanent damage to the radiation-induced auditory pathways had developed.

It is known that an awareness of the mean and maximum cochlear doses is important in the assessment of cochlear toxicity. Studies on GKR have reported that the risk of hearing loss is increased in VS patients who received >4 Gy to the cochlea [3,23,24]. In our study, the mean cochlear dose was 3.1 Gy, and the mean maximum cochlear dose was 5.8 Gy. In patients with functional hearing, the maximum cochlear dose was kept below 4 Gy as much as possible. In our study, there was a weak correlation between increased ADC values at ipsilateral MGB and cochlear radiation dose. This weak connection may suggest that increased ADC values in MGB are unrelated to the cochlear radiation dose.

Recently, diffusion spectrum imaging (DSI), a new diffusion MRI technique, has been developed to non-invasively detect complex white matter pathway architecture and structural changes in fiber pathways in order to overcome the limitations of DTI. Brain reconstruction via DSI shows neural connectivity, such as associative, association, and projection fibers. DSI reconstructs fiber lines with a much higher resolution than DTI and is considered to accurately display transition, winding, and interrupted and small fibers [25-27]. Omnidirectional fiber orientation analysis can be avoided using DSI. The general fractional anisotropy (GFA) value is the main quantitative parameter of DSI, representing the directional consistency of water molecule diffusion. DSI is increasingly used nowadays, especially in neuropsychiatric, neurodegenerative, and white matter diseases [25-27].

There are some limitations to our study. Firstly, ROIs were selected for evaluation. When placing ROIs at different locations in the auditory pathways, the problems caused by the partial volume effect can be considered. Secondly, the DTI data of the auditory pathways were not analyzed by tract-based fully automated systems.

## Conclusions

High ADC values ​​in the contralateral HG after radiosurgery may indicate microstructural alterations such as a decrease in the number of axons and an increase in extracellular fluid due to damage to the myelin sheath. Radiation exposure doses to the brainstem and cochlea are the most important factors that can cause microstructural damage to the auditory pathways. We believe that more attention should be paid to prevent the harmful effects of radiation on the auditory pathways when planning radiosurgery. Long-term follow-up and comprehensive DSI studies in the future are needed to analyze in detail the effects of radiosurgery on the auditory pathways in patients with VS and overcome the limitations of DTI.
